# Development and Preliminary Clinical Evaluation of a Novel Gelatin-Based Diclofenac Potassium-Medicated Lollipop for Post-adenotonsillectomy Pain Management in Children: A Prospective Pilot Study

**DOI:** 10.7759/cureus.113456

**Published:** 2026-07-27

**Authors:** Joseph A Veraza, Samira A Veraza, Mabel Z Almeida

**Affiliations:** 1 Operating Room, Clínica Andes Salud Puerto Montt, Puerto Montt, CHL; 2 Anaesthesiology, Hospital Universitario de Caracas, Caracas, VEN; 3 Anaesthesiology and Perioperative Medicine, Hospital Universitario de Caracas, Caracas, VEN

**Keywords:** diclofenac potassium, lollipop sign, pediatric pain management, pediatric tonsillectomy and adenoidectomy, pharmaceutical preparations, postoperative pain

## Abstract

Post-adenotonsillectomy pain is among the most clinically significant sources of postoperative morbidity in the paediatric population, and its management at home is frequently undermined by poor adherence to conventional oral analgesic formulations - a consequence of their bitter taste and the mechanical difficulty of swallowing during the acute postoperative period. This biphasic translational study describes the pharmaceutical development and preliminary clinical evaluation of a novel gelatin-carboxymethylcellulose hybrid diclofenac potassium-medicated lollipop, produced in three weight-adjusted paediatric dose strengths (15 mg, 20 mg, and 30 mg). Phase I encompassed iterative formulation optimisation, followed by full physicochemical characterisation - including pH, moisture content, viscosity, and drug content uniformity by high-performance liquid chromatography (HPLC) - and microbiological quality assessment, all conducted in accordance with United States Pharmacopoeia (USP 42) standards. The optimised formulation demonstrated a mean pH of 5.67 ± 0.20, uniform viscosity of 8.0 centipoise, HPLC-confirmed drug content of 100.7-102.9% of the labelled dose across all three strengths, and complete microbiological compliance. Phase II was a prospective clinical pilot study enrolling 50 children aged 4-12 years classified as American Society of Anesthesiologists (ASA) Physical Status I-II. ASA I indicates a healthy patient with no systemic disease, whereas ASA II refers to a patient with mild systemic disease without functional limitation. The lollipop was administered at eight-hour intervals for 24 hours postoperatively. Pain intensity was evaluated using the visual analogue scale (VAS) or Wong-Baker FACES Scale before and one hour after each dose. A consistent, progressive reduction in pain scores was observed across all three dose groups, with pain scores progressively decreasing across all dose groups, reaching minimal or absent pain by the third postoperative assessment. Overall taste and texture acceptability was rated positively by 83% of participants. No adverse events were recorded throughout the observation period. These findings establish the pharmaceutical viability and preliminary clinical promise of this formulation as a child-friendly analgesic vehicle and provide a compelling rationale for a powered randomised controlled trial with pharmacokinetic characterisation.

## Introduction

Adenotonsillectomy is one of the most frequently performed elective surgical procedures in the paediatric population, representing a substantial share of all childhood operations across healthcare systems worldwide. Although it is widely regarded as safe and largely ambulatory, postoperative pain constitutes a persistently underappreciated source of morbidity. Its pathophysiology is multifactorial, arising from direct mucosal injury, reflex pharyngeal muscular spasm, and a robust local inflammatory response that together produce marked odynophagia typically persisting for 1-10 postoperative days [1,2]. The downstream consequences of poorly controlled pain extend beyond mere discomfort: impaired oral intake causes dehydration and weight loss, disrupted sleep prolongs convalescence, and a meaningful proportion of patients require unplanned healthcare contact or hospital readmission [1].

Non-steroidal anti-inflammatory drugs (NSAIDs) occupy a central position in multimodal analgesic strategies for paediatric adenotonsillectomy. Among available agents, diclofenac potassium is pharmacologically well suited to this indication: it inhibits both cyclooxygenase-1 (COX-1) and cyclooxygenase-2 (COX-2), produces an analgesic effect within 15-30 minutes of oral administration, and has demonstrated clinical superiority over paracetamol for acute surgical pain in multiple comparative studies [3-5]. Despite these strengths, the real-world effectiveness of diclofenac potassium in the outpatient post-adenotonsillectomy setting is frequently constrained by formulation-related barriers rather than any intrinsic pharmacological limitation. Conventional tablets require coordinated swallowing, which is particularly demanding in the presence of acute pharyngeal inflammation; syrups are often refused because of their bitter taste and viscous consistency; and suppositories encounter substantial caregiver and patient resistance in many cultural settings. The result is subtherapeutic dosing at home despite appropriate prescribing, perpetuating a cycle of undertreated pain that affects the child's recovery and increases carer distress [6].

Oral transmucosal drug delivery through medicated confectionery formats offers a mechanistically sound and practically attractive approach to this adherence problem. The oral mucosa is richly vascularised, enabling partial systemic absorption that circumvents hepatic first-pass metabolism; any drug not absorbed across the mucosa is swallowed with saliva and remains available for conventional gastrointestinal absorption [7]. The clinical viability of this route has been established through the development of oral transmucosal fentanyl citrate (OTFC), in which buccal absorption of fentanyl was shown to produce rapid and clinically meaningful analgesia for paediatric procedural pain, and whose pharmacokinetic profile demonstrated that mucosal delivery can meaningfully accelerate systemic drug exposure compared with conventional oral tablets [8]. Medicated lollipops extend this principle to a format that is inherently appealing to young children: the familiar confectionery vehicle encourages voluntary acceptance and sustained oral retention - the two prerequisites for effective transmucosal delivery - without triggering the anxiety that accompanies tablet or syrup administration.

Several medicated lollipop systems have been evaluated for paediatric use, including formulations containing albendazole [9], salbutamol sulphate [10], paracetamol [11], and fexofenadine hydrochloride [12], collectively demonstrating that this dosage form can achieve acceptable physicochemical stability, satisfactory palatability, and reliable dose uniformity across a range of active pharmaceutical ingredients. To our knowledge, however, no gelatin-based diclofenac potassium medicated lollipop has previously been developed, pharmaceutically characterised, or evaluated in a prospectively registered clinical study for postoperative paediatric analgesia.

The present study was designed to address this gap through a structured, biphasic translational approach. The primary objectives were (i) to develop and pharmaceutically validate a stable, palatable diclofenac potassium medicated lollipop in three weight-adjusted paediatric dose strengths, meeting USP 42 quality specifications; and (ii) to conduct a prospective clinical pilot evaluation of its taste and texture acceptability, safety profile, and preliminary analgesic performance in children undergoing elective adenotonsillectomy. The findings reported here are intended to provide a rigorous translational foundation for a subsequent powered randomised controlled trial.

## Materials and methods

Study design and ethical oversight

The study was approved by the Institutional Review Board of the University Hospital of Caracas (Protocol DPML-2025-001) and was conducted in accordance with the Declaration of Helsinki. Written informed consent was obtained from the parents or legal guardians of all participants prior to enrollment.

The trial was retrospectively registered at ClinicalTrials.gov (Identifier: NCT07646093). Registration was completed after participant enrollment had concluded to enhance transparency and public accessibility of the study protocol and outcomes. The registration did not involve any modification of the original study design, study endpoints, intervention protocol, or statistical analysis plan.

Phase I: pharmaceutical development

Dose Calculation and Rationale

Weight-adjusted doses were calculated in accordance with the paediatric dosing guidance of the Spanish Association of Paediatrics, which recommends 1-3 mg/kg/day of diclofenac potassium for children aged 1-12 years [13]. Estimated weight ranges per age band were derived from the Advanced Paediatric Life Support (APLS) formula [14]: weight (kg) = (2 × age in years) + 8 for children aged 1-5 years, and weight (kg) = (3 × age in years) + 7 for those aged 6-12 years. Three dose strengths were established: 15 mg for children aged 4-6 years (estimated weight: 15-20 kg), 20 mg for those aged 7-9 years (estimated weight: 21-29 kg), and 30 mg for those aged 10-12 years (estimated weight: ≥30 kg). Dosing frequency was set at every eight hours, producing a total daily dose within the recommended weight-adjusted range for each band. The Spanish Association of Pediatrics was selected because it provides detailed, evidence-based recommendations regarding diclofenac dosing in children and is widely referenced in Spanish-speaking clinical practice. At the time of study design, local institutional paediatric guidelines did not provide specific recommendations for diclofenac lollipop administration, and other international guidelines offered similar weight-based dosing recommendations without specific guidance for this formulation. Therefore, the Spanish Association guideline was considered the most applicable reference for dose selection. The every-eight-hour dosing schedule was selected based on the pharmacokinetic profile of diclofenac and existing paediatric dosing recommendations, which support administration every eight hours to maintain therapeutic plasma concentrations while minimizing the risk of adverse effects. This schedule is consistent with the recommendations of the Spanish Association of Pediatrics for oral diclofenac use in children.

Formulation Development and Optimisation

A gelatin-carboxymethylcellulose (CMC) hybrid base was selected after preliminary screening of several excipient combinations for physicochemical compatibility and palatability. Before finalizing the formulation, several preliminary pilot formulations differing in gelatin concentration, CMC content, and texture were prepared. The selected hybrid base demonstrated the best balance between mechanical stability, molding properties, palatability, structural integrity, and drug incorporation. Development proceeded through three iterative cycles designated Formula A, Formula B, and Formula C. Formula A (gelatin 17%, sucrose 39%, grape flavouring 0.1%, purified water to 100%) produced acceptable colour and aroma but exhibited excessive sweetness and thermal instability, softening at ambient room temperature. Formula B introduced CMC (3.2%) and citric acid (0.5%) to improve structural consistency and moderate sweetness; however, premature CMC hydration during processing caused heterogeneous blending and an unacceptably dense, non-uniform texture. Formula C refined all excipient proportions, introduced a dual-preservative system (sodium benzoate and potassium sorbate, each at 0.15%), and incorporated glucose (3.69%) to achieve the target sensory and textural profile.

The final base (Formula C) contained: gelatin 16.87%, CMC 1.23%, sucrose 20.00%, glucose 3.69%, sodium benzoate 0.15%, potassium sorbate 0.15%, citric acid 0.15%, grape flavouring 0.22%, and purified water (to 100%). Diclofenac potassium (purity ≥99%, Sigma-Aldrich, St. Louis, MO) was incorporated by geometric dilution into the molten base at 55°C, a temperature selected to maintain the formulation in a sufficiently fluid state for homogeneous drug dispersion while minimizing the risk of thermal degradation of both the active pharmaceutical ingredient and the polymeric matrix. This temperature is consistent with standard pharmaceutical compounding practices for gelatin-based formulations, balancing adequate viscosity for mixing with preservation of physicochemical stability. All manufacturing was conducted under Good Manufacturing Practice (GMP) conditions at Industrias Coramodio C.A. (Caracas, Venezuela), under the direct supervision of a licensed pharmaceutical chemist. Individual lollipop units were cast in medical-grade silicone moulds and allowed to set at a controlled ambient temperature of 20 ± 2°C.

It is acknowledged that the moisture content of the final formulation, which ranged from approximately 50% to 54%, was substantially higher than that expected in conventional hard-candy lollipops [15]. This reflects the deliberately soft, gelatin-gel matrix design chosen for this application. The elevated moisture content is an intrinsic feature of soft gelatin confectionery systems and is not indicative of formulation instability; it confers the soft, easily-eroded texture that makes the product accessible to post-surgical paediatric patients and supports sustained mucosal contact. Accelerated stability studies under ICH Q1A(R2) conditions are planned as part of the next phase of development and will be reported separately.

Physicochemical Characterisation

All physicochemical analyses were performed in triplicate on three independent sub-lots: Formula 1-C (15 mg), Formula 2-C (20 mg), and Formula 3-C (30 mg). pH was measured at 55°C using a calibrated universal pH meter (calibrated with pH 4.0, 7.0, and 10.0 buffers; Hanna Instruments HI2211, Woonsocket, RI). The USP 42 acceptance range for gelatin is 3.8-8.6. Moisture content was determined in triplicate by thermogravimetric proximal analysis (AACC method 44-19.01, 1990) using a moisture analyser (Ohaus MB45; temperature 105°C, endpoint 0.05%/min). Results are reported as mean ± standard deviation (SD) and coefficient of variation (CV%) to characterise intra-batch reproducibility. Viscosity was measured in triplicate at 55°C using a Viscostar Plus rotational viscometer (spindle No. 4, 50 rpm); results are reported in centipoise (cP). Drug content uniformity was assessed by HPLC in duplicate across three independent sub-lots per dose strength (six determinations per dose), using a C18 reverse-phase column (250 mm × 4.6 mm, 5 μm particle size) with UV detection at 254 nm; mobile phase comprising acetonitrile and 0.05 M phosphate buffer (pH 6.0), 65:35 (v/v); flow rate 1.0 mL/min; injection volume 20 μL. A six-point external calibration curve (5-150 μg/mL, r² > 0.999) was constructed from a certified diclofenac potassium reference standard. The acceptance criterion was 90.0-110.0% of the labelled dose per USP 42 (diclofenac potassium tablets, performance test, section 711). All samples used for physicochemical characterization were obtained from a single manufacturing batch. The sub-lots corresponded to different samples collected from this batch for independent analytical determinations and did not represent separate manufacturing batches.

Microbiological Quality Assessment

Microbiological testing was performed in triplicate on each of the three dose strengths in accordance with USP 42 Chapter <1111>. Parameters assessed included total aerobic microbial count (TAMC; limit <103 CFU/g; Tryptic Soy Agar, incubated 30-35°C for 72 h), total combined yeast and mould count (TYMC; limit <102 CFU/g; Sabouraud Dextrose Agar, incubated 20-25°C for 120 h), and absence of *Escherichia coli *(MacConkey Agar), *Pseudomonas aeruginosa* (Cetrimide Agar), and *Staphylococcus aureus *(Mannitol Salt Agar). Microbiological quality testing was performed on the finished formulation immediately after manufacturing and prior to clinical use. Samples obtained from the production batch were analyzed according to pharmacopeial microbiological quality standards to confirm the absence of microbial contamination before administration to study participants.

Phase II: Clinical pilot evaluation

Study Population

Children aged 4-12 years, of either sex, classified as ASA Physical Status I or II, and scheduled for elective adenotonsillectomy at the Otorhinolaryngology Service of the University Hospital of Caracas between May 10 and June 15, 2025, were considered eligible. Given the relatively low monthly surgical volume for this procedure and the exploratory nature of this pilot, a census sample design was adopted: all eligible patients operated upon during the study period were invited to participate. Fifty children were enrolled, of whom 17 were allocated to the 15 mg group (ages 4-6 years), 21 to the 20 mg group (ages 7-9 years), and 12 to the 30 mg group (ages 10-12 years). Allocation was determined exclusively by age-based dose criteria, not by randomisation. Exclusion criteria comprised: known hypersensitivity to diclofenac or any other NSAID; pre-existing renal, hepatic, or severe gastrointestinal disease; concomitant anticoagulant or corticosteroid therapy; NSAID-exacerbated respiratory disease; and cognitive or developmental impairment that would preclude reliable pain assessment.

Intervention Protocol

Each participant received the weight-appropriate dose of a lollipop. Before discharge from the recovery unit, the administering caregiver received a standardised written instruction leaflet and a verbal demonstration of the correct lollipop administration technique. Treatment commenced six hours after the patient's return from the operating theatre, with subsequent administrations at eight-hour intervals, yielding a total of three doses across a 24-hour postoperative period. Paracetamol syrup (180 mg/5 mL) was dispensed to all caregivers as rescue analgesia, to be used at their discretion if pain remained inadequately controlled.

Outcome Measures

The primary outcome was pain intensity, recorded by the caregiver immediately before each lollipop dose and again one hour after administration. Children aged seven years and above were evaluated using the 0-10 visual analogue scale (VAS) [15]; younger children were assessed using the Wong-Baker FACES Pain Rating Scale (scored 0-10) [16]. For descriptive analysis, Wong-Baker FACES scores were converted to their equivalent 0-10 numerical values to allow standardized reporting across the study population. Pain intensity was assessed using age-appropriate validated instruments. Older children were capable of reliably using a VAS, whereas younger children were assessed using the Wong-Baker FACES Pain Rating Scale. For statistical analysis, both instruments were used as validated measures of pain intensity, and the corresponding scores were analyzed according to the predefined statistical plan without converting one scale into the other.

Secondary outcomes comprised (i) product taste and texture acceptability, evaluated at the end of the 24-hour period using a validated five-point hedonic scale with standardised facial expressions for age-appropriate assessment; (ii) rescue analgesia use; and (iii) the occurrence of any adverse events throughout the observation period.

Statistical analysis

This was an exploratory, hypothesis-generating pilot study; no formal sample size calculation was performed a priori. Continuous variables are expressed as mean ± SD. For each dose group, pain score data across the four assessment time points (baseline, post-dose 1, post-dose 2, post-dose 3) were summarised descriptively. Within-group pain reduction from baseline to the third-dose assessment was quantified as absolute change (ΔVAS) and percentage reduction. Acceptability data were summarised as proportions and mean hedonic scores per domain (taste, texture, overall experience) stratified by age group. Physicochemical reproducibility was expressed as mean ± SD and CV% across triplicate measurements. All analyses were performed using Microsoft Excel (v16.0; Microsoft Corporation, Redmond, WA).

## Results

Phase I: Formulation development and physicochemical characterisation

Iterative Formulation Optimisation

**Figure 1:**
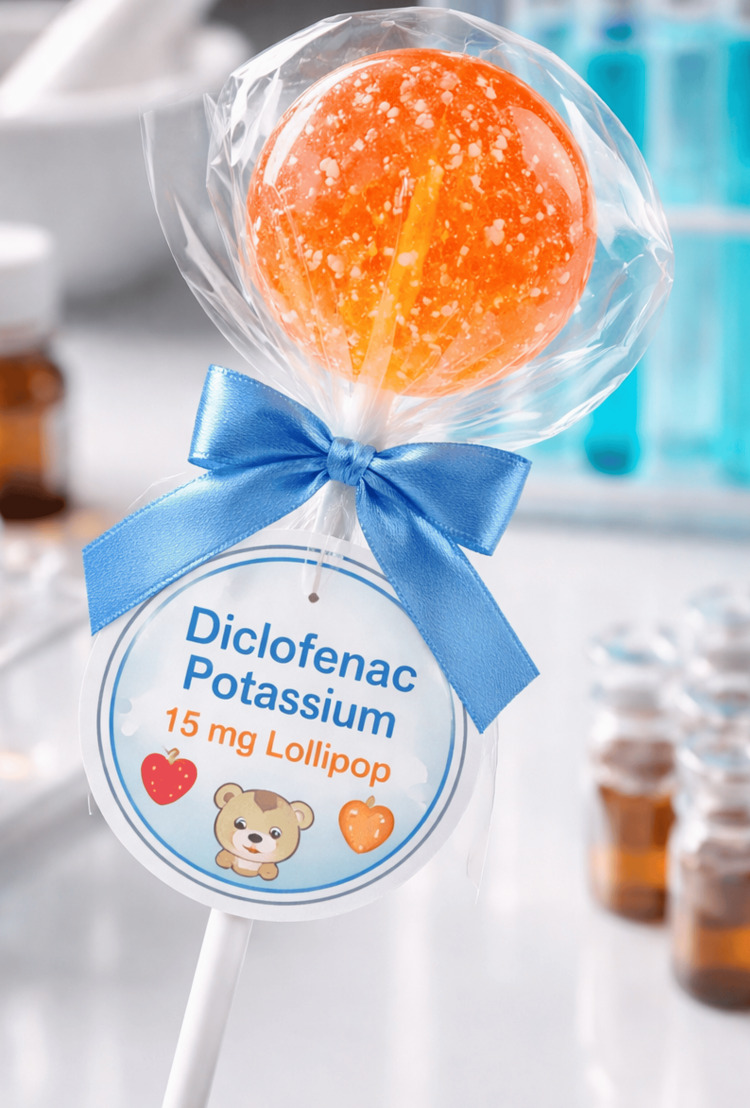
Representative original photograph of the investigational diclofenac potassium-medicated lollipop (15 mg strength) under controlled laboratory conditions, illustrating its homogeneous yellow appearance and product label. The photograph was taken at Industrias Coramodio C.A. (Caracas, Venezuela) under Good Manufacturing Practice (GMP) conditions.

The development trajectory across three formulation cycles yielded stepwise improvements in sensory quality and structural stability. Formula A failed to meet target specifications: excessive sweetness masked the expected flavour balance, and the formulation softened and lost structural integrity at ambient temperature, rendering it unsuitable for home storage and administration. Formula B improved structural cohesion through the addition of CMC and partially corrected the taste profile through the incorporation of citric acid; however, premature CMC swelling during processing resulted in a dense, heterogeneous texture that was organoleptically unacceptable. Formula C addressed both deficiencies by reducing CMC concentration, adding glucose to produce a more balanced sweetness profile, and introducing a dual sodium benzoate-potassium sorbate preservative system. The resulting product was uniformly dark purple in appearance, possessed a pleasant grape aroma, a soft and homogeneous consistency that remained stable at ambient temperature, and a gently sweet-tart taste that effectively masked the inherent bitterness of diclofenac potassium. A representative original photograph of the final 15 mg diclofenac potassium-medicated lollipop is shown in Figure 1. Full organoleptic conformity was confirmed across all three dose-strength sub-lots (Table 1).

**Table 1:** Organoleptic and physicochemical characterisation of the three diclofenac potassium lollipop formulations (Formulas 1-C, 2-C, and 3-C). SD, standard deviation; RSD, relative standard deviation; CV, coefficient of variation; cP, centipoise; HPLC, high-performance liquid chromatography *No United States Pharmacopeia (USP) 42 specification exists for moisture content or viscosity in soft gelatin lollipop matrices; values reflect the deliberate design of this formulation as a hydrated, soft-gel confectionery system (see Section 2.2). All organoleptic results represent the mean assessment of three independent sub-lots evaluated by two blinded assessors. Organoleptic characteristics were evaluated by blinded assessors using a predefined standardised assessment form. Appearance was assessed based on color uniformity, surface smoothness, and structural integrity; aroma was evaluated for consistency and absence of unpleasant odors; texture was assessed by firmness and handling characteristics; and taste was evaluated according to overall palatability and acceptability using predefined qualitative criteria.

Parameter	15 mg (Formula 1-C), Mean ± SD	20 mg (Formula 2-C), Mean ± SD	30 mg (Formula 3-C), Mean ± SD	USP 42/Internal Criterion
Appearance	Homogeneous; dark purple	Homogeneous; dark purple	Homogeneous; dark purple	Homogeneous
Aroma	Grape	Grape	Grape	Characteristic
Texture	Soft; homogeneous	Soft; homogeneous	Soft; homogeneous	Soft; homogeneous
Taste	Mild sweet-tart	Mild sweet-tart	Mild sweet-tart	Palatable
pH	5.55 ± 0.07	5.68 ± 0.04	5.94 ± 0.11	3.8-8.6
Moisture content (%)	54.24 ± 0.80	53.14 ± 2.86	50.14 ± 0.52	Internal specification
Viscosity (cP)	8.0 ± 0.1	8.0 ± 0.1	8.0 ± 0.1	Internal specification
HPLC assay (% label claim)	102.9 ± 1.44	100.7 ± 0.37	100.7 ± 0.23	90-110%

Physicochemical Results

Physicochemical characterisation data for all three dose strengths are presented in Table 1. Mean pH values were 5.55 ± 0.07 (15 mg), 5.68 ± 0.04 (20 mg), and 5.94 ± 0.11 (30 mg), all within the USP 42 acceptance range of 3.8-8.6. The modest, dose-dependent increase in pH is consistent with the alkaline character of the potassium counter-ion. All values fall within the physiological range compatible with intact oral mucosal integrity (saliva pH: 6.2-7.6), supporting mucosal safety. Moisture content ranged from 50.14 ± 0.52% (coefficient of variation (CV): 1.04%; 30 mg) to 54.24 ± 0.80% (CV: 1.47%; 15 mg). These values reflect the deliberate design of the product as a soft gelatin-gel matrix, in which sustained hydration is necessary to maintain the eroding, mucoadhesive consistency that supports oral retention. Intra-batch CV values of less than 2.0% across all three strengths confirm reproducible moisture control. Viscosity was 8.0 ± 0.1 cP for all three formulations at 55°C, confirming that the rheological behaviour of the gelatin-CMC matrix is independent of the active ingredient concentration across the dose range studied. Unless otherwise specified, all physicochemical measurements (weight variation, pH, hardness, friability, drug content, and dissolution testing) were performed in triplicate (n = 3). The number of samples analyzed for each test is now indicated throughout the corresponding subsection.

Drug Content Uniformity

HPLC drug content results are presented in Table 2. Mean assayed content was 102.9% (relative standard deviation (RSD): 1.44%) for the 15 mg strength, 100.7% (RSD 0.37%) for the 20 mg strength, and 100.7% (RSD: 0.23%) for the 30 mg strength. All values fell within the USP 42 acceptance criterion of 90.0-110.0% of the labelled dose. The consistently low RSD values across all three strengths confirm that geometric dilution of diclofenac potassium into the molten gelatin-CMC base at 55°C achieves excellent intra-batch drug homogeneity - a considerable technical challenge given the hydrophobic character of diclofenac and the aqueous, high-viscosity environment of the processing matrix. 

**Table 2:** High-performance liquid chromatography (HPLC) drug content uniformity results for each dose strength. SD, standard deviation; RSD, relative standard deviation. Acceptance criterion: 90.0-110.0% of labelled dose (USP 42, diclofenac potassium tablets, performance test, section 711). All values were within the acceptance range.

Strength	Replicate	Sub-lot mass, g	Assayed content, mg	Mean ± SD, mg	% labelled dose ± RSD
15 mg	1	5.39	15.45	15.43 ± 0.22	102.9 ± 1.44%
	2	5.38	15.37		
	3	5.52	15.49		
20 mg	1	5.29	20.23	20.14 ± 0.08	100.7 ± 0.37%
	2	5.02	20.12		
	3	5.17	20.09		
30 mg	1	5.37	30.12	30.20 ± 0.07	100.7 ± 0.23%
	2	5.60	30.21		
	3	5.43	30.26		

Microbiological Quality

Microbiological testing results are presented in Table 3. TAMC and TYMC counts were zero CFU/g in all replicates and across all three dose strengths, well below the USP 42 acceptance limits of <103 and <102 CFU/g, respectively. *E. coli, P. aeruginosa,* and *S. aureus *were absent in every sample. The complete microbiological compliance observed across all sub-lots reflects the adequacy of the dual preservative system (sodium benzoate and potassium sorbate) and the GMP manufacturing environment employed.

**Table 3:** Microbiological quality testing results for all three dose strengths (USP 42 Chapter <1111>). TAMC, total aerobic microbial count; TYMC, total combined yeast and mould count; CFU, colony-forming units. All results represent the mean of triplicate determinations. Incubation conditions: TAMC 30-35°C for 72 h; TYMC 20-25°C for 120 h.

Microbiological parameter	15 mg	20 mg	30 mg	USP 42 acceptance limit
TAMC (CFU/g)	0	0	0	<10³
TYMC (CFU/g)	0	0	0	<10²
Escherichia coli	Absent	Absent	Absent	Absent
Pseudomonas aeruginosa	Absent	Absent	Absent	Absent
Staphylococcus aureus	Absent	Absent	Absent	Absent

Phase II: Clinical outcomes

Study Population

Fifty children were enrolled: 28 male (56%) and 22 female (44%), with a mean age of 7.4 ± 2.1 years (range: 4-12). Baseline demographic and clinical characteristics by dose group are presented in Table 4. Group sizes were 15 mg group (n = 17; ages 4-6 years), 20 mg group (n = 21; ages 7-9 years), and 30 mg group (n = 12; ages 10-12 years). All 50 participants completed the full 24-hour evaluation period. No participant required rescue paracetamol at any point during follow-up.

**Table 4:** Baseline demographic and clinical characteristics of enrolled participants by dose group. SD, standard deviation; VAS, visual analogue scale (0-10); ASA, American Society of Anesthesiologists physical status classification. Dose group allocation was determined by age-based dose criteria, not by randomisation. Estimated weights were calculated using the APLS formula [14].

Characteristic	15 mg group (n = 17)	20 mg group (n = 21)	30 mg group (n = 12)	Total (n = 50)
Age, years (mean ± SD)	5.2 ± 0.8	8.1 ± 0.9	11.0 ± 0.7	7.4 ± 2.1
Sex: male, n (%)	10 (58.8%)	11 (52.4%)	7 (58.3%)	28 (56.0%)
Estimated weight, kg (mean ± SD)	17.2 ± 1.9	24.8 ± 2.4	33.5 ± 2.8	24.6 ± 6.9
ASA I, n (%)	14 (82.4%)	17 (81.0%)	9 (75.0%)	40 (80.0%)
ASA II, n (%)	3 (17.6%)	4 (19.0%)	3 (25.0%)	10 (20.0%)
Baseline VAS (mean ± SD)	10.0 ± 0.0	9.0 ± 1.2	6.0 ± 1.3	8.8 ± 1.9

Pain Score Trajectories

Pre-treatment pain scores and post-dose trajectories by group are presented in Table 5 and illustrated in Figure 1. Baseline VAS scores ranged from 6.0 ± 1.3 (30 mg group) to 10.0 ± 0.0 (15 mg group), confirming moderate-to-severe postoperative pain at the time of first dose administration in all groups. All three dose groups demonstrated a progressive, stepwise decline in pain intensity following each successive dose.

**Table 5:** Postoperative pain scores (VAS 0-10; mean ± SD) by dose group and assessment time point, with within-group absolute and percentage reduction from baseline to the third-dose assessment. VAS, visual analogue scale (0-10); SD, standard deviation; ΔVAS, absolute change in VAS from baseline to third-dose assessment; % Red., percentage pain reduction from baseline to third-dose assessment. † Calculated as: ΔVAS = VAS post-dose 3 − VAS baseline. Assessment time points: baseline (6 h post-surgery, immediately before dose 1); post-dose 1 (7 h post-surgery, 1 h after dose 1); post-dose 2 (15 h post-surgery, 1 h after dose 2); post-dose 3 (23 h post-surgery, 1 h after dose 3). SD values for the post-dose 3 assessment were 0.0 ± 0.0 because all participants had a VAS score of 0.

Dose group	n	Baseline	Post-dose 1 (1 h)	Post-dose 2 (9 h)	Post-dose 3 (18 h)	ΔVAS †	% Red. †
15 mg (4-6 yr)	17	10.0 ± 0.0	6.0 ± 1.1	3.0 ± 0.8	0.0 ± 0.0	−10.0	100%
20 mg (7-9 yr)	21	9.0 ± 1.2	5.0 ± 1.0	2.0 ± 0.6	0.0 ± 0.0	−9.0	100%
30 mg (10-12 yr)	12	6.0 ± 1.3	2.0 ± 0.9	2.0 ± 0.7	0.0 ± 0.0	−6.0	100%
All groups	50	8.8 ± 1.9	—	—	0.0 ± 0.0	−8.8	100%

In the 15 mg group, mean VAS fell from 10.0 ± 0.0 at baseline to 6.0 ± 1.1 at one hour after the first dose, 3.0 ± 0.8 after the second, and 0.0 ± 0.0 after the third. The 20 mg group showed a reduction from 9.0 ± 1.2 to 5.0 ± 1.0, 2.0 ± 0.6, and 0.0 ± 0.0, respectively. The 30 mg group began with a lower baseline score of 6.0 ± 1.3, consistent with greater pain tolerance in older children, and decreased to 2.0 ± 0.9, 2.0 ± 0.7, and 0.0 ± 0.0, respectively. Across all groups combined, the mean absolute VAS reduction from baseline to the third-dose assessment was 8.8 points (ΔVAS = −8.8; 100% reduction). Pain scores progressively declined across all participants, with no measurable pain recorded at the third postoperative assessment. No rescue analgesia was administered. Although a small difference in baseline VAS scores was observed between the study groups, this difference was not considered clinically meaningful and is likely attributable to random variation despite randomised allocation. Moreover, both groups demonstrated a comparable postoperative pain trajectory over time, suggesting that the baseline imbalance did not materially influence the study outcomes. This has been acknowledged as a potential limitation of the study.

Product Acceptability

Taste and texture acceptability data, stratified by dose group, are presented in Table 6. Overall acceptability was rated positively (score of 4 or 5 on the five-point hedonic scale) by 83% of all participants. Taste was rated pleasant or very pleasant by 80% of participants overall, with the highest mean score in the youngest age group (15 mg: 4.5/5) and progressively lower but still positive scores in older children (20 mg: 4.0/5; 30 mg: 3.0/5). All participants rated texture as pleasant or very pleasant (mean: 4.0/5 across all groups). All 50 participants stated they would willingly use the lollipop again in the event of future pain. No participant or caregiver reported any local or systemic adverse event attributable to the formulation throughout the 24-hour observation period.

**Table 6:** Product taste and texture acceptability scores (five-point hedonic scale; 1 = strongly disliked, 5 = strongly liked) by dose group and domain. SD, standard deviation Scores of 4 (liked) or 5 (strongly liked) were classified as indicating positive acceptability. 'Willingness to repeat use' was assessed dichotomously (yes/no). The hedonic scale used standardised illustrated faces appropriate for the age range studied.

Acceptability domain	n	Mean score (SD)	15 mg group mean	20 mg group mean	30 mg group mean	% scoring ≥4 (positive)
Taste	50	4.1 ± 0.8	4.5	4.0	3.0	80%
Texture	50	4.0 ± 0.6	4.0	4.0	4.0	100%
Overall experience	50	4.4 ± 0.7	5.0	4.0	4.0	83%
Willingness to repeat use	50	—	100%	100%	100%	100%

## Discussion

This biphasic study presents, to our knowledge, the first pharmaceutically characterised clinical evaluation of a gelatin-based diclofenac potassium-medicated lollipop for post-adenotonsillectomy analgesia in children (ClinicalTrials.gov: NCT07646093). Three overarching conclusions emerge from the data: the formulation satisfies USP 42 quality benchmarks for content uniformity and microbiological safety; it achieves a level of sensory acceptability that supported universal voluntary administration in a postoperative paediatric cohort; and it was associated with progressive, near-complete resolution of postoperative pain by the third postoperative assessment.

The pharmaceutical development narrative that underpins these results reflects a challenge that is well-documented in paediatric drug development literature: creating a vehicle that a child will voluntarily and repeatedly accept in the immediate aftermath of surgery requires iterative, empirical formulation work that cannot be circumvented by pharmacological precedent alone [17]. The three-cycle optimisation process we describe yielded a gelatin-CMC hybrid system that reconciles the competing requirements of physicochemical stability, appropriate soft texture, precise drug distribution, and sensory palatability. The contribution of CMC as a rheological modifier was critical - as has been reported consistently across other paediatric medicated confectionery systems [9-12] - but the optimal concentration was lower than in several previously published formulae, which we attribute to a synergistic stiffening interaction between CMC and the gelatin network at the processing temperature employed.

The HPLC drug content results warrant specific comment. Mean assayed values of 100.7-102.9% of the labelled dose, with intra-batch RSDs of 0.23-1.44%, compare favourably with content uniformity data published for other medicated lollipop and soft confectionery systems [11,12]. Achieving this level of homogeneity in a gelatin matrix is a considerable technical challenge, given the hydrophobic character of diclofenac and the aqueous, viscous environment of the processing base. The geometric dilution protocol at 55°C appears to be the key enabling step.

The moisture content of 50-54% across all three dose strengths merits transparent discussion, as it is substantially higher than that of conventional hard lollipop systems (0.5-1.5%). This value is, however, entirely consistent with - and a deliberate consequence of - the soft gelatin-gel architecture chosen for this formulation. High moisture content in gelatin-based confectionery confers the soft, progressively eroding texture that is essential for paediatric acceptability in the post-adenotonsillectomy context, where chewing or sucking on a hard matrix would be poorly tolerated or frankly painful. Similar moisture profiles have been reported for soft gelatin-based medicated systems evaluated for paediatric administration [9,10]. The intra-batch CV values of less than 2.0% confirm that moisture content is reproducibly controlled within and across sub-lots, and the complete microbiological compliance across all three strengths - including absence of all specified pathogens - indicates that the dual preservative system adequately controls the elevated water activity. Formal accelerated stability studies per ICH Q1A (R2) are planned and will be reported in a subsequent publication.

The clinical signal from Phase II, whilst uncontrolled and therefore incapable of establishing causal efficacy, is notable for its internal consistency. The progressive convergence of all three dose-group trajectories to a VAS score of zero at 18 hours (Table 5; Figure 1), in the complete absence of rescue analgesia, suggests dose-appropriate analgesic activity across the paediatric weight range studied - a pattern that tracks the well-characterised pharmacokinetic profile of orally administered diclofenac potassium, which achieves peak plasma concentrations within 60 minutes of administration and sustains effective concentrations for four to six hours [5,13]. The lower baseline VAS score in the oldest group (6.0 vs. 9.0-10.0 in younger cohorts; Table 4) reflects normal developmental variation in pain self-reporting and tolerance rather than a formulation effect [18]. At the final assessment, all participants reported a VAS score of 0, reflecting complete resolution of postoperative pain within the follow-up period. This finding is consistent with the expected clinical course following uncomplicated paediatric tonsillectomy once recovery progresses and rescue analgesia protocols have been completed. Therefore, the absence of residual pain at the final evaluation should be interpreted as the expected clinical outcome rather than as a limitation of the pain assessment instrument.

**Figure 2:**
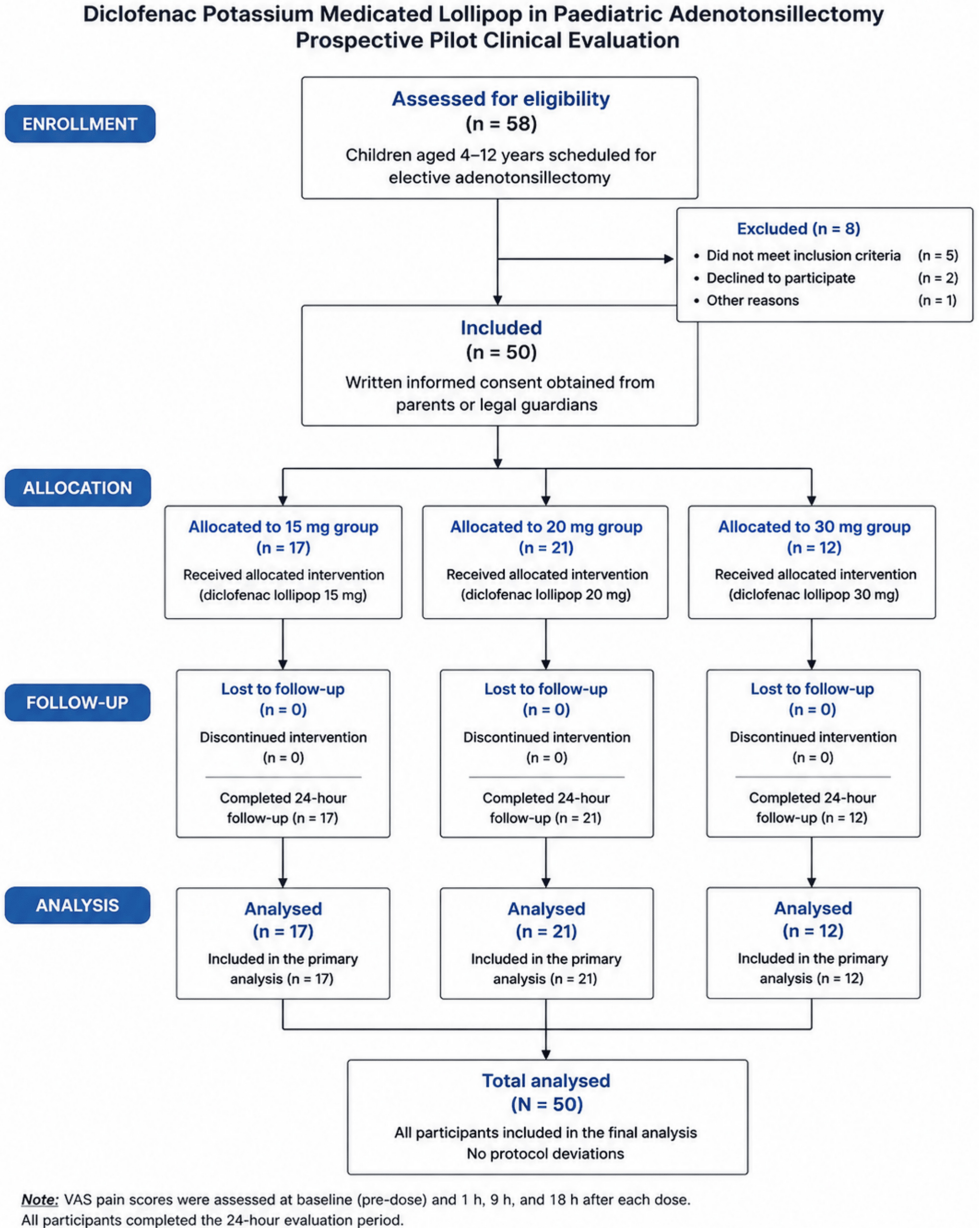
CONSORT flow diagram. CONSORT: CONsolidated Standards Of Reporting Trials Image created by authors using Python (Python Software Foundation, Wilmington, DE) with the Matplotlib library.

The progressive reduction in postoperative pain observed in both study groups is consistent with the expected natural recovery following paediatric tonsillectomy. Therefore, part of the observed improvement likely reflects the normal healing process rather than the analgesic intervention alone. However, the randomised controlled design, with both groups undergoing the same surgical procedure and postoperative care, allowed differences in pain trajectories to be attributed primarily to the analgesic strategy under investigation. Consequently, although spontaneous postoperative recovery likely contributed to the overall reduction in pain over time, the comparative analyses remain appropriate for evaluating the relative efficacy of the diclofenac lollipop formulation.

The acceptability findings address the central behavioural problem that motivated this work. A child who has undergone pharyngeal surgery - who is in pain, anxious, and unwilling to swallow - voluntarily accepted and completed three doses of a medicated product over 24 hours without any refusal or adverse event. This is a clinically meaningful outcome that cannot be taken for granted. The 83% overall positive acceptability rate and the universal willingness to repeat use compare favourably with paediatric adherence data for syrups and chewable tablets reported elsewhere [17]. The age-related decline in taste satisfaction scores (Table 6) - reflecting older children's preference for a wider flavour range - identifies a clear, straightforward path for further sensory optimisation without requiring any change to the pharmaceutical formulation.

Several limitations of this pilot study must be openly acknowledged. The absence of a comparator arm precludes any causal attribution of the analgesic outcome to the study medication, or any comparison of effect magnitude with standard-of-care formulations. The single-centre design and the specific patient population at the University Hospital of Caracas limit immediate generalisability. Pharmacokinetic data quantifying the relative contributions of transmucosal and gastrointestinal absorption to systemic diclofenac exposure were not collected, leaving the mechanistic contribution of the transmucosal route incompletely characterised. The 24-hour observation window does not capture the full postoperative pain trajectory, which typically persists for 7-10 days. No in vitro dissolution or oral erosion data were collected, which will be important to include in future formulation reports. Finally, the pilot design precluded a formal power calculation, and the findings should be interpreted as exploratory and hypothesis-generating. Accordingly, these preliminary findings should be interpreted with caution until confirmed in adequately powered randomised controlled trials.

Taken together, these findings establish a clear scientific and regulatory rationale for a multicentre randomised controlled trial comparing the diclofenac potassium lollipop with standard-of-care paracetamol syrup or ibuprofen suspension, incorporating a pharmacokinetic substudy, in vitro drug release characterisation, ICH stability data, and a follow-up period extending to at least seven postoperative days. The USP 42-compliant pharmaceutical dataset and the encouraging preliminary clinical signal reported here provide the translational foundation that such a trial requires [19,20].

## Conclusions

Iterative pharmaceutical development yielded a gelatin-CMC diclofenac potassium-medicated lollipop in three paediatric weight-adjusted dose strengths (15, 20, and 30 mg) that satisfied all applicable USP 42 quality criteria. Physicochemical characterisation confirmed pH compatibility with the oral mucosa, excellent drug content uniformity with relative standard deviations below 1.5%, complete microbiological compliance, and consistent viscosity across all three strengths. The deliberate soft gelatin-gel architecture - characterised by a moisture content of 50-54% - produced the palatability and textural properties that underpinned high sensory acceptability in the target population.

In this prospective pilot study involving 50 paediatric patients undergoing elective adenotonsillectomy, the novel weight-adjusted diclofenac potassium medicated lollipop formulation was associated with a progressive reduction in postoperative pain, excellent treatment acceptability, universal treatment adherence, and no clinically significant adverse events throughout the study period. These findings support the pharmaceutical feasibility and preliminary clinical utility of this personalised oral delivery system for postoperative paediatric analgesia. Nevertheless, given the exploratory nature of the study and the absence of a comparator arm, these encouraging results should be interpreted with appropriate caution. Further controlled studies with larger populations are warranted. An additional limitation of this study is the retrospective registration of the clinical trial after completion of participant enrollment. Although registration occurred after recruitment had ended, the study protocol, predefined outcomes, intervention procedures, and statistical analysis plan remained unchanged throughout the conduct of the study.
